# Comment on “the IS*6* family, a clinically important group of insertion sequences including IS*26*” by Varani and co-authors

**DOI:** 10.1186/s13100-021-00257-9

**Published:** 2022-01-03

**Authors:** Ruth M. Hall

**Affiliations:** School of Life and Environmental Sciences, The University of Sydney, Sydney, NSW 2006 Australia

**Keywords:** IS*26* family, Insertion sequence IS*26*, Insertion sequences IS*431* and IS*257*, Insertion sequence IS*1216*, Insertion sequences IS*1006*, IS*1008* and IS*1006*/*1008*, Targeted conservative cointegrate formation, Copy-in cointegrate formation, Translocatable unit (TU), Pseudo-compound transposon

## Abstract

The insertion sequence IS*26* has long been known to play a major role in the recruitment of antibiotic resistance genes into the mobile resistance gene pool of Gram-negative bacteria and IS*26* also plays a major role in their subsequent broad dissemination. Related IS, IS*431/257* and IS*1216* are important in the same roles in Gram positive bacteria. However, until recently the properties of IS*26* movement that could potentially explain this ability had not been explored. A much needed insight has come from our recent demonstration that IS*26* uses a novel targeted mechanism that is conservative. The targeted conservative mechanism is much more efficient than the known replicative mechanism, which is now more accurately called copy-in. A recent review “The IS*6* family, a clinically important group of insertion sequences including IS*26*” by Varani, He, Siguier, Ross and Chandler published in Mobile DNA has substantially misrepresented the recent studies on the targeted conservative mechanism and at the same time incorrectly implied that any mechanism established for IS*26* can be assumed to apply to a range of IS that are at best very distantly related. A few of the most important issues are examined in this comment. Readers are advised to consult the original literature to check facts before drawing firm conclusions.

## Background

We recently discovered that, in addition to cointegrate formation via the known copy-in (formerly replicative) route, IS*26* can generate cointegrates between any pair of DNA molecules that each include an IS using a novel, targeted mechanism that is completely conservative [1]. This finding was extended to some other members of the IS*26* family, namely IS*257* and IS*1216* [2] and IS*1006*, IS*1008* and a naturally-occurring l IS1*006*/IS*1008* hybrid [3]. We have now confirmed that the end-products of both IS*26*-mediated reactions are exclusively cointegrates [4]. Hence, homologous recombination is needed to resolve the cointegrates and complete the movement of these IS to a new location.

## Main text

The presentation of the findings about the capabilities of IS*26* arising from the recent body of work conducted in my laboratory in the recent review “The IS*6* family, a clinically important group of insertion sequences including IS*26*” by Varani, He, Siguier, Ross and Chandler [5] raises concerns with respect to inaccuracies and misrepresentation. Indeed, Varani and co-authors claim that there is “an absence of formal proof” for the existence of the targeted conservative mechanism. As I believe that our in depth experimental approach to the reactions that occur in vivo has produced a level of information that would normally be considered to amount to formal proof, I recommend that the original references should be read before their view is accepted.

We have explored aspects of the requirements of the targeted conservative reaction in more detail [3, 6] and the speculative mechanism for the targeted conservative route presented by Varani et al. in Fig. 11 is of particular concern because it is not consistent with those experimental findings. In fact, the 2017 study [6] that established that only one end of each participating IS*26* is needed for the targeted conservative reaction to occur was not cited. A model that is consistent with the currently available data can be found in Fig. 5 in [3]. However, further work is still needed.

In addition, we have shown experimentally that the targeted conservative mechanism can generate the IS*26*-bounded pseudo-compound transposons and the overlapping pseudo-compound transposon configurations found in many multiply antibiotic resistant Gram-negative pathogens [1, 7]. This route involves a non-replicating circular intermediate containing a single IS*26* that was named a translocatable unit (TU). However, Varani et al. also question the existence of TU, even though they clearly can be formed de novo at very low frequency via the copy-in mechanism in adjacent deletion mode, and this is the first step in the likely route to initial resistance gene recruitment. IS*26*-mediated insertion of such TU by either mechanism then generates a pseudo-compound transposon. TU can also arise readily by homologous recombination between any directly-oriented pair of IS*26*s such as those flanking pseudo-compound transposons. Hence, pseudo-compound transposons can change their location via a TU formed by homologous recombination followed by IS*26* action [8]. This is in clear contrast to the claim in the review that pseudo-compound transposon movement can only occur via cointegrate formation between two replicons followed by resolution via homologous recombination.

In addition, we have identified the group of IS that share most similarity to IS*26* in their transposases and terminal inverted repeats allowing the inference that they are most likely to share the dual mechanistic capabilities of IS*26*. We refer to the members of the group of six clades most closely related to IS*26* (see Figs. 1 and 3 in [9]) as the IS*26* family [9]. In contrast, Varani et al. prefer a much larger family that they call the IS*6* family. However, then they have claimed, via use of “IS*6* family members” or equivalent when describing the properties of IS*26*-based pseudo-compound transposons and our experimental data, that our findings are applicable to all members of the IS*6* family, as they define it. However, our data were obtained only with IS*26* or with a few related IS (IS*257*/IS*431*, IS*1216*, IS*1006*, IS*1008* and an IS*1006/1008* hybrid) that are members of the IS*26* family as we define it [9]. We have been unable to find any experimental evidence for an activity of any member of the additional very distantly related groups that are included in their IS*6* family, and none was cited. Hence, to the best of our knowledge, the claim that these more distantly related IS have the same mechanistic capabilities as IS*26* and relatives, which is implicit in their assignment of these IS to the same family, is not supported by any evidence.

## Conclusions

The review by Varani et al. contains a number of inaccuracies. Most notably, evidence for targeted, conservative cointegration by IS*26* and related elements is substantially stronger than Varani et al. imply. In addition, to date, there is no data supporting the extension of this mechanism to IS elements beyond the IS*26* family as we previously defined it. Readers are advised to base their conclusions on the primary literature.

## References

[1] Harmer CJ, Moran RA, Hall RM (2014). Movement of IS*26*-associated antibiotic resistance genes occurs via a translocatable unit that includes a single IS*26* and preferentially inserts adjacent to another IS*26*. mBio..

[2] Harmer CJ, Hall RM (2020). IS*26* family members IS*257* and IS*1216* also form cointegrates by copy-in and targeted conservative routes. mSphere..

[3] Harmer CJ, Hall RM (2021). Targeted conservativecointegrate formation mediated by IS*26* family members requires sequence identity at the reacting end. mSphere..

[4] Harmer CJ, Hall RM (2021). IS26 cannot move alone. J Antimicrob Chemother.

[5] Varani A, He S, Siguier P, Ross K, Chandler M (2021). The IS*6* family, a clinically important group of insertion sequences including IS*26*. Mob DNA.

[6] Harmer CJ, Hall RM (2017). Targeted conservative formation of cointegrates between two DNA molecules containing IS*26* occurs via strand exchange at either IS end. Mol Microbiol.

[7] Harmer CJ, Hall RM (2016). IS*26*-mediated formation of transposons carrying antibiotic resistance genes. mSphere..

[8] Harmer CJ, Pong CH, Hall RM (2020). Structures bounded by directly-oriented members of the IS*26* family are pseudo-compound transposons. Plasmid..

[9] Harmer CJ, Hall RM (2019). An analysis of the IS*6*/IS*26* family of insertion sequences: IS it a single family?. Microb Genom.

